# Comparison of rehabilitation outcomes for transcatheter versus surgical aortic valve replacement as redo procedure in patients with previous cardiac surgery: Evidence based on 11 observational studies

**DOI:** 10.1097/MD.0000000000027657

**Published:** 2021-11-12

**Authors:** Guobin Wang, Xuefeng Li, Zhaojie Zhang, Jige Dong

**Affiliations:** a Rehabilitation Division Treatment Department, Wang Jing Hospital of China Academy of Chinese Medical Science, Beijing, China; b Department of Vascular Surgery, Wang Jing Hospital of China Academy of Chinese Medical Science, Beijing, China; c Department of Spinal Surgery, Wang Jing Hospital of China Academy of Chinese Medical Science, Beijing, China.

**Keywords:** aortic valve replacement, mortality, prior cardiac surgery, transcatheter aortic valve replacement

## Abstract

**Background:**

Currently, the number of severe aortic stenosis (AS) patients with a history of prior cardiac surgery (PCS) has increased. Both transcatheter aortic valve replacement (TAVR) and traditional surgical aortic valve replacement (sAVR) are effective therapy for AS. However, PCS increases the risk of adverse outcomes in patients undergoing aortic valve replacement. Thus, this meta-analysis was designed to comparatively evaluate the impact of PCS on clinical outcomes between TAVR and sAVR.

**Methods:**

A systematic search of PubMed, Embase, Cochrane Library, and Web of Science up to February 1, 2021 was conducted for relevant studies that comparing TAVR and sAVR for severe AS patients with a history of PCS. The primary outcome was the non-inferiority of TAVR and sAVR in mortality. The secondary outcomes were the other clinical outcomes. Two reviewers assessed trial quality and extracted the data independently. All statistical analyses were performed using the standard statistical procedures provided in Review Manager 5.2.

**Results:**

A total of 11 studies including 8852 patients were identified. The pooled results indicated that there was no difference in 30-day, and 1-year all-cause mortality between TAVR and sAVR. No significant difference was also observed in total follow-up and cardiovascular mortality between TAVR and sAVR. However, subgroup analysis revealed significantly higher 1-year all-cause mortality (OR 1.92; 95% CI 1.05–3.52; *P* = .04) and total follow-up mortality (OR 2.28; 95% CI 1.09–4.77; *P* = .03) in TAVR than sAVR for patients with a history of coronary artery bypass graft, aortic valve replacement, and mitral valve reconstruction. In addition, TAVR experienced higher pacemaker implantation than sAVR. However, compared with sAVR, TAVR experienced shorter length of stay (MD –3.18 days; 95% CI –4.78 to –1.57 days) and procedural time (MD –172.01 minutes; 95% CI –251.15 to –92.88) respectively. TAVR also lead to much less bleeding than sAVR.

**Conclusions:**

Our analysis shows that TAVR as a redo procedure was equal to sAVR in mortality for severe AS patients with PCS, especially coronary artery bypass graft. We agree the advantage of TAVR as a redo procedure for patients with a history of PCS. Patients receiving TAVR experienced rapid recovery, shorter operation time and less bleeding, without increasing short and long term mortality.

## 1 Introduction

At present, degenerative aortic valve disease, as one of the most frequent valvular heart disease with a severity ranging from aortic sclerosis slowly progressing to symptomatic severe aortic stenosis (AS), usually requires aortic valve replacement.^[1]^ In patients older than 75 years, AS is present in 12.4% of the population, with severe forms in 3.4% of the elderly.^[2]^ Currently, though surgical aortic valve replacement (sAVR) was a traditional effective method for patients with symptomatic severe AS, transcatheter aortic valve replacement (TAVR) as an effective and convenient intervention has been adopted extensively.

According to the European and American guidelines, symptomatic severe AS requires sAVR or TAVR, with a mean survival of 2 to 3 years in the absence of these procedures.^[3,4]^ TAVR is increasingly used in high and more recently in intermediate-risk population, studies evaluating now the indication even in low-risk population.^[5–8]^ The 2017 American Heart Association Valvular Guidelines^[9,10]^ have given TAVR a Class I recommendation for these patients at high or prohibitive surgical risk. For those at intermediate risk, TAVR is considered a reasonable alternative to sAVR,^[7,11]^ with a Class IIA recommendation in the American Heart Association guidelines.^[9,10]^

A history of prior cardiac surgery (PCS) may increase the risk of surgery to a large extent. Currently, the number of severe AS patients with a history of PCS has increased. Both TAVR and traditional sAVR are effective therapy for AS. However, the efficacy and safety of TAVR have not yet been well evaluated and its non-inferiority compared with traditional sAVR still lack sufficient evidence for AS patients with a history of PCS. Thus, this meta-analysis was designed to comparatively evaluate the impact of PCS on clinical outcomes between TAVR and sAVR.

## 2 Methods

### 2.1 Search strategy and study selection

A systematic search of PubMed, Embase, Cochrane Library, and Web of Science up to February 1, 2021 was conducted for relevant studies using a search strategy developed by a medical information specialist that involved controlled vocabulary and keywords related to our research question (e.g., “prior cardiac surgery,” “previous cardiac surgery,” “history of cardiac surgery,” “aortic stenosis,” “valvular heart disease,” “aortic valve disease”; “transcatheter aortic valve replacement,” “transcatheter aortic valve implantation,” “surgical aortic valve replacement,” “surgical aortic valve implantation,” “TAVR,” “TAVI,” “SAVR,” “SAVI”; “survival,” “outcome,” “prognosis,” “mortality,” “complication”). The search strategy was limited to English language articles. Two assessors independently screened the titles and abstracts of each study. When a relevant study was identified, its full text was obtained for further evaluation. The full text of related references was also obtained for review.

The present study was approved by the Ethics Committee of Wang Jing Hospital of China Academy of Chinese Medical Science.

### 2.2 Criteria for considering studies

We included studies if they met the following criteria: studies that compared TAVR with sAVR; AS patients with a history of cardiac surgery; studies in which the relevant outcomes of both TAVR and sAVR groups were assessed; and patients who were diagnosed with severe aortic disease.

Studies were excluded if they met the following criteria: experimental trial on animals or a non-human study; study population included patients with other diseases that would affect outcomes; study reported in the form of an abstract, letter, editorial, expert opinion, review, or case report; or lack of sufficient data or failure to meet the inclusion criteria.

### 2.3 Quality assessment and data extraction

Two assessors receiving normative training beforehand independently evaluated the quality of all the included studies using the 9-star Newcastle-Ottawa Scale.^[12]^ The scores were judged according to the 3 aspects of Newcastle-Ottawa Scale of evaluation: selection, comparability, and outcome between the case group and control group. In order to observe the bias of our included studies better, the risk of bias for each studies and the risk of bias across all studies were evaluated and shown with figures generated by RevMan 5.2 software.^[13]^

Baseline characteristics and outcomes from the included studies were extracted using a standardized extraction form. Key study characteristics including country, sample size, mean age, PCS type, surgical risk, follow-up time and primary endpoint, were extracted. Data were extracted by 1 reviewer and then examined for accuracy and completeness by a second reviewer.

### 2.4 Data synthesis and statistical methods

The data of comparable outcomes between TAVR and sAVR were combined-analyzed, using the standard statistical procedures provided in RevMan 5.2.^[13]^ Dichotomous data were measured with risk ratio (OR) and continuous variable data were measured with mean difference (MD). The heterogeneity between studies was evaluated by the chi-square-based *Q* statistical test,^[14]^ with *P*_h_ value and *I*^2^ statistic, ranging from 0% to 100%, to quantify the effect of heterogeneity. *P*_h_ ≤ .10 was deemed to represent significant heterogeneity,^[15]^ and pooled estimates were estimated using a random-effect model (the DerSimonian and Laird method^[16]^). On the contrary, if statistical study heterogeneity was not observed (*P*_h_ > .10), a fixed effects model (the Mantel–Haenszel method^[17]^) was used. The effects of outcome measures were considered to be statistically significant if pooled RRs with 95% CI did not overlap with 1 or pooled MDs with 95% CI did not overlap with 0. Additionally, publication bias was assessed by Begg test. If the shape of funnel plots revealed no obvious evidence of asymmetry, we considered that there was no obvious publication bias.

This work has been reported in line with Preferred Reporting Items for Systematic Reviews and Meta-Analyses (PRISMA)^[18]^ and Assessing the methodological quality of systematic reviews (AMSTAR) Guidelines.^[19]^

## 3 Results

### 3.1 Included studies, study characteristics, and quality assessment

**Figure 1:**
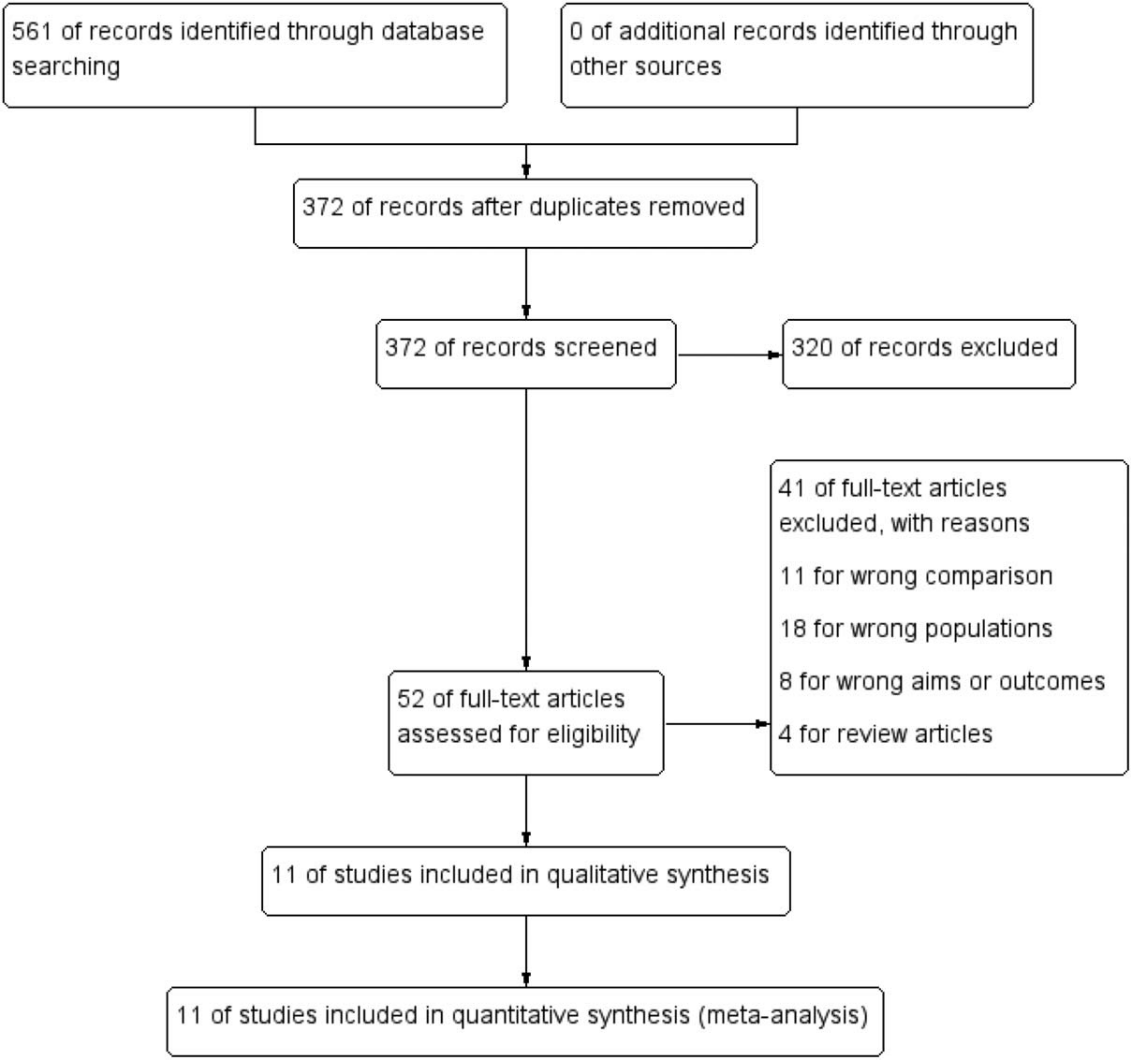
Flow diagram of literature search and selection of included studies for meta-analysis.

At the beginning of the search, a total of 561 records of citations were obtained; 372 of records were reviewed further after duplicates were removed. Via screening the titles and abstracts, 320 studies were excluded preliminarily and then 52 studies were chosen to get full texts for further evaluation. After reading the full texts, 41 studies were excluded further (11 studies for wrong comparison, 18 for wrong population, 8 for wrong aims or outcomes, and 4 for review articles). Eventually, 11 observational studies^[20–30]^ (N = 8852 participants) were included in this systematic review and meta-analysis. Of these studies, 3 studies included AS patients with multi-type PCS with coronary artery bypass graft (CABG), aortic valve replacement (AVR), mitral valve reconstruction.^[20,26,27]^ The others included AS patients with only CABG.^[21–25,28–30]^ The detailed search process and summary of studies are shown in the study flow diagram (Fig. 1). The other characteristics of each study are shown in Table 1.

**Table 1:** The characteristics of included studies in this meta-analysis.

		Sample size		Age (mean ± SD, range)			Surgical risk (mean ± SD)			
Study ID	Country	TAVR	sAVR	TAVR	sAVR	PCS type	TAVR	sAVR	Follow-up time	Primary endpoint
Chen S (2018)	Multicountry	245	264	78.5 ± 7.0	79.4 ± 6.5	CABG	6.1 ± 2.1^†^	6.1 ± 2.0^†^	2 years	All-cause death and disabling stroke
Conte JV (2016)	USA	115	111	82.0 ± 5.8	81.0 ± 5.9	CABG	7.3 ± 2.7^†^	8.0 ± 3.5^†^	1 year	All-cause mortality at 1 year
Greason KL (2014)	Georgia	148	140	80.7 ± 7.0	82.3 ± 6.2	CABG	11.8 ± 3.3^†^	12 ± 3.1^†^	Up to 2 years	Death of any cause at 1 year
Gupta T (2018)	USA	3380	3380	80.7 ± 7.2	73.6 ± 8.7	CABG	NR	NR	NR	All-cause, in-hospital mortality
Jegaden O (2012)	France	13	10	76 ± 11	76 ± 6	CABG	25 ± 14^‡^	25 ± 16^‡^	Mean 1.2 years	The 1-year survival
Nguyen TC (2014)	Georgia	107	148	79.8 ± 7.9	72.5 ± 8.8	CABG	11.8 ± 6.6^†^	7.1 ± 5.6^†^	Up to 2 years	Short-term and midterm outcomes
Papadopoulos N (2014)	Germany	52	167	82 ± 5	72 ± 9	CABG, AVR, MVR	11 ± 4^†^	9 ± 2^†^	4 ± 2 years	The incidence of major AEs including death and permanent neurologic complications
Reardon MJ (2019)	Multicountry	136	137	76.9 ± 6.5	76.6 ± 6.5	CABG	5.0 ± 1.6^†^	5.2 ± 1.7^†^	1-year	All-cause mortality or disabling stroke
Stortecky S (2011)	Switzerland	40	40	78.2 ± 6	70.6 ± 8	CABG	7.6 ± 7^†^	6.3 ± 6^†^	6 months	Perioperative and mid-term clinical outcome
Wendt D (2015)	Germany	62	51	78.7 ± 5.9	71.1 ± 10.8	CABG, AVR, MVR	12.1 ± 10^†^	7.1 ± 5.2^†^	1-year	In-hospital mortality, defined as all causes of death within 30 days
Wilbring M (2013)	Germany	53	53	78.1 ± 5.5	77.6 ± 2.7	CABG, AVR, MVR	8.8 ± 3.72^∗^	8.16 ± 4.4^∗^	245 ± 323 days	Clinical and mid-term outcomes

AEs = adverse events, AVR = aortic valve replacement, CABG = coronary artery bypass graft, EuroSCORE = the European System for Cardiac Operative Risk, MVR = mitral valve reconstruction, NR = no report, PCS = prior cardiac surgery, sAVR = surgical aortic valve replacement, STS = Society of Thoracic Surgeons, TAVR = transcatheter aortic valve replacement.

∗ Displayed as EuroSCORE II.

† Displayed as STS score (%).

‡ Displayed as Logistic EuroSCORE (%).

**Figure 2:**
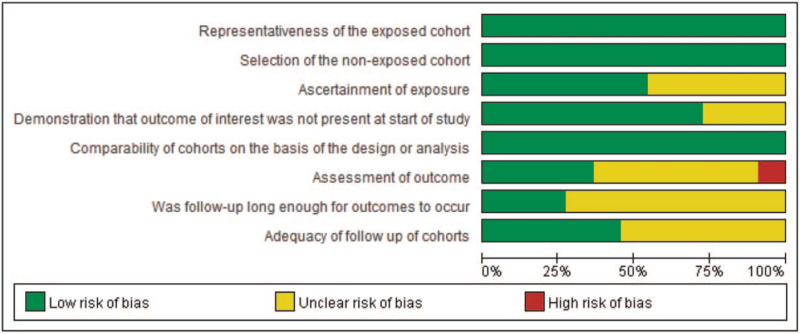
Risk of bias graph: review authors’ judgements about each risk of bias item presented as percentages across all included studies.

**Figure 3:**
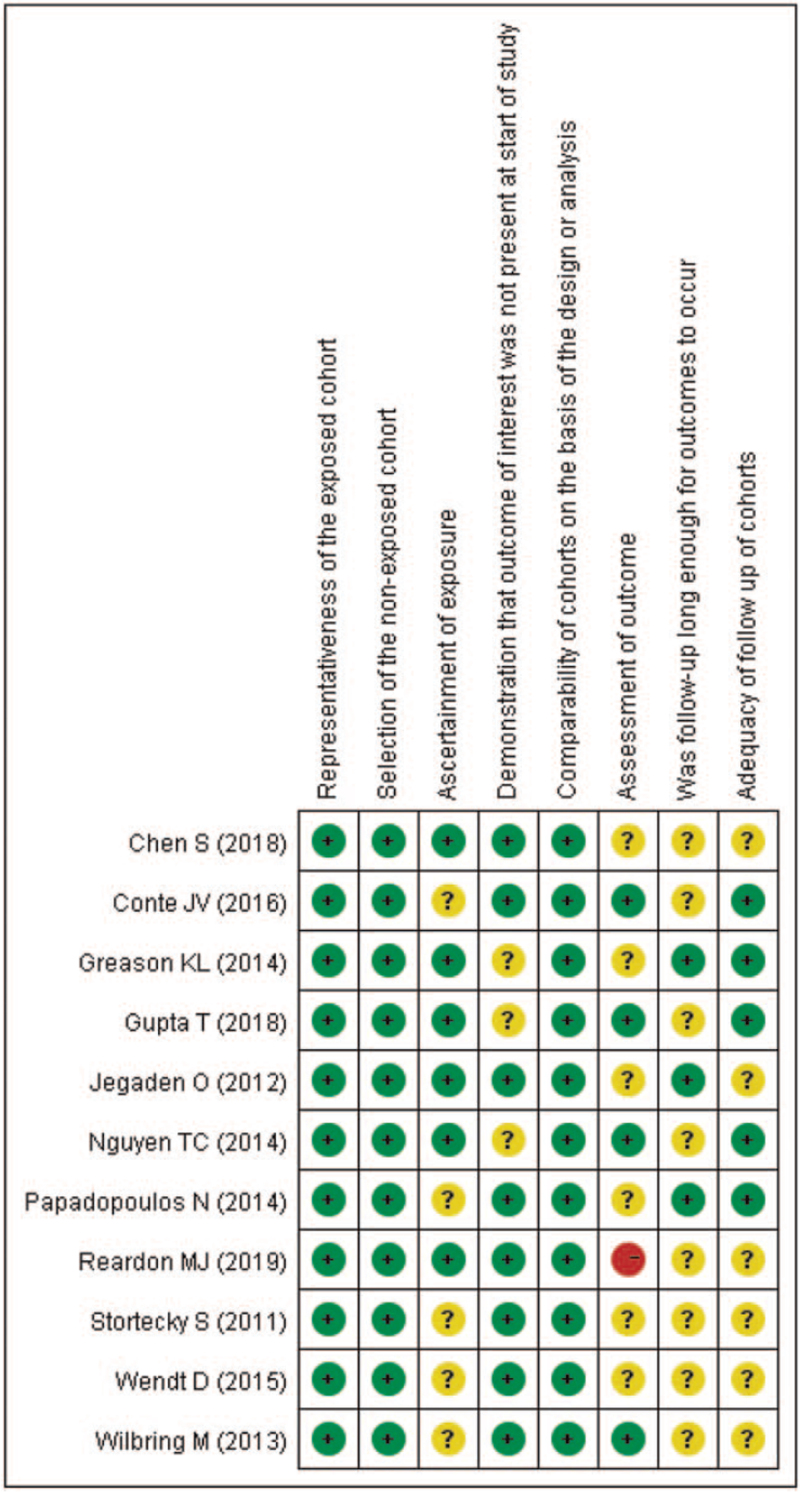
Risk of bias summary: review authors’ judgements about each risk of bias item for each included study.

Risk-of-bias graphs were generated to further identify the risk of bias of the including studies. The risk of bias for each study was presented as percentages across all included studies in Fig. 2, and the risk of bias item for each included study is displayed in Fig. 3. The risk of bias graphs indicated generally good quality in “selection,” including items of “representativeness of the exposed cohort” and “selection of the non-exposed cohort.” Besides, all studies experienced low risk of bias for “comparability.” Unclear risk of bias was mainly observed in “outcomes” including “assessment of outcomes,” “adequacy of follow-up of cohorts,” and “follow up enough for outcomes” issues. High risk of bias was only observed in Reardon et al^[29]^ about “assessment of outcomes.”

### 3.2 Comparison between TAVR and sAVR regarding to baseline characteristics

We compared the baseline characteristics of both TAVR and sAVR groups. As Table 2 showing, the age of PCS patients in TAVR group were older than sAVR (MD 3.58 year; 95% CI 0.81–6.53; *P* = .01; 8852 pts). In addition, TAVR groups showed higher surgery risk than sAVR groups, with a pooled MD of Society of Thoracic Surgeons score of 0.99 (95% CI 0.13–1.85; *P* = .02; 1963 pts). There was no difference between TAVR and sAVR groups in NYHA ≥III (OR 0.91; 95% CI 0.56–1.47), Logistic the European system for cardiac operative risk (%) (MD 3.86; 95% CI –1.58–9.31), Serum Cr (mg/dL) (MD –0.12; 95% CI –0.38–0.14), chronic obstructive pulmonary disease (OR 1.42; 95% CI 0.92–2.19), previous myocardial infarction (OR 1.14; 95% CI 0.55–2.38), previous cerebral vascular disease (OR 1.0; 95% CI 0.73–1.38), peripheral vascular disease (OR 1.43; 95% CI 0.76–2.72), diabetes mellitus (OR 1.22; 95% CI 0.72–2.07), and sex (OR 0.99; 95% CI 0.71–1.38) respectively.

**Table 2:** The pooled baseline characteristics results of comparison between TAVR and sAVR.

		Pooled results			Heterogeneity		
Subgroups	No. of patients	MD/OR	95% CI	*P* value	*I* ^2^	*P*_h_ value	Analytical effect model
Age, yr	8852	MD 3.58	0.81, 6.53	.01	98%	<.00001	Random-effect model
STS score (%)	1963	MD 0.99	0.13, 1.85	.02	89%	<.00001	Random-effect model
NYHA ≥ III	577	OR 0.91	0.56, 1.47	.70	0%	.56	Fixed effects model
Logistic EuroSCORE (%)	617	MD 3.86	−1.58, 9.31	.61	72%	.01	Random-effect model
Serum Cr, mg/dL	543	MD –0.12	−0.38, 0.14	.35	0%	.83	Fixed effects model
COPD	849	OR 1.42	0.92, 2.19	.11	52%	.10	Random-effect model
Previous MI	368	OR 1.14	0.55, 2.38	.72	55%	.13	Random-effect model
Previous CVD	849	OR 1.0	0.73, 1.38	.99	1%	.39	Fixed effects model
PVD	849	OR 1.43	0.76, 2.72	.27	79%	.002	Random-effect model
DM	849	OR 1.22	0.72, 2.07	.46	70%	.02	Random-effect model
Male	849	OR 0.99	0.71, 1.38	.94	0%	.92	Fixed effects model

CI = confidence intervals, COPD = chronic obstructive pulmonary disease, Cr = creatinine, CVD = cerebral vascular disease, DM = diabetes mellitus, MD = mean difference, MI = myocardial infarction, OR = odds ratio, PM = pacemaker, PVD = peripheral vascular disease.

### 3.3 Comparison between TAVR and sAVR regarding to mortality

**Figure 4:**
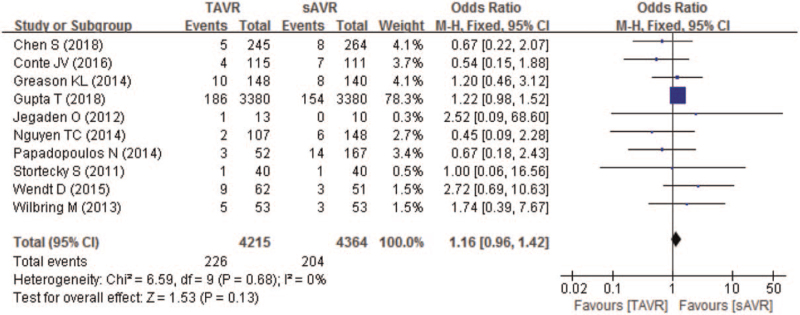
Forest plot of comparison between TAVR and sAVR regarding to 30-day all-cause mortality. sAVR = surgical aortic valve replacement, TAVR = transcatheter aortic valve replacement.

Ten studies compared 30-day all-cause mortality of patients with severe AS between TAVR and sAVR groups. As Fig. 4 showing, though the 30-day all-cause mortality was higher in TAVR (5.4%) than sAVR group (4.7%), no significant difference was found (OR 1.16; 95% CI 0.96–1.42; *P* = .13; 8579 pts). In addition, no significant difference in 30-day all-cause mortality between TAVR and sAVR groups was found in patients with a history of only CABG (OR 1.15; 95% CI 0.94–1.41) or multiple cardiac surgeries (OR 1.4; 95% CI 0.67–2.93) (Table 3).

**Table 3:** The subgroup analysis of mortality between TAVR and sAVR according to PCS type.

		Pooled results			Heterogeneity		
Subgroups	No. of patients	OR	95% CI	*P* value	*I* ^2^	*P*_h_ value	Analytical effect model
30- day all-cause mortality							
CABG	8141	1.15	0.94, 1.41	.18	0%	.66	Fixed effects model
CABG, AVR, MVR	438	1.4	0.67, 2.93	.37	11%	.32	Fixed effects model
1-year all-cause mortality							
CABG	1301	1.02	0.76, 1.39	.88	37%	.17	Fixed effects model
CABG, AVR, MVR	438	1.92	1.05, 3.52	.04	0%	.55	Fixed effects model
Total follow-up mortality							
CABG	1381	1.11	0.84, 1.46	.46	40%	.14	Fixed effects model
CABG, AVR, MVR	332	2.28	1.09, 4.77	.03	0%	.48	Fixed effects model
CV mortality							
CABG	594	0.80	0.45, 1.44	.46	30%	.24	Fixed effects model

AVR = aortic valve replacement, CABG = coronary artery bypass graft, CI = confidence intervals, MVR = mitral valve reconstruction, OR = odds ratio.

**Figure 5:**
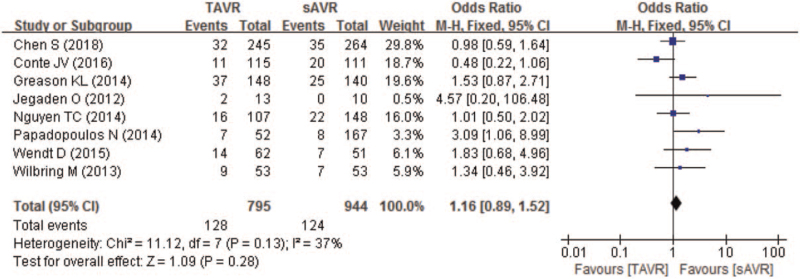
Forest plot of comparison between TAVR and sAVR regarding to 1-year all-cause mortality. sAVR = surgical aortic valve replacement, TAVR = transcatheter aortic valve replacement.

Similarly, compared with sAVR, TAVR as a redo procedure also showed non-inferiority in 1-year all-cause mortality (16.1% of TAVR vs 13.1% of sAVR), with a pooled OR of 1.16 (95% CI 0.89–1.52; *P* = .28; 1739 pts) (Fig. 5). In addition, 1-year all-cause mortality of CABG patients in TAVR group was equal to sAVR group (OR 1.02; 95% CI 0.76–1.39). However, for patients previously received multiple cardiac surgeries, subgroup analysis indicated higher 1-year all-cause mortality in TAVR than sAVR (OR 1.92; 95% CI 1.05–3.52) (Table 3).

**Figure 6:**
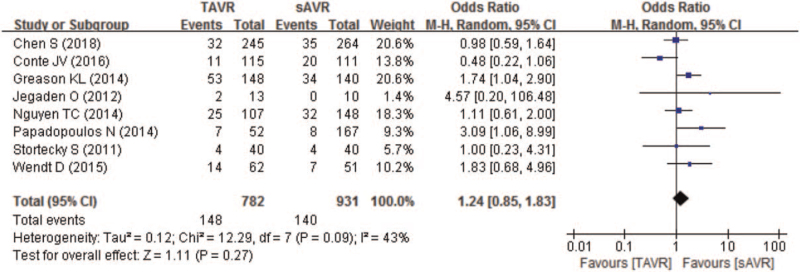
Forest plot of comparison between TAVR and sAVR regarding to total follow-up mortality. sAVR = surgical aortic valve replacement, TAVR = transcatheter aortic valve replacement.

Eight studies compared total follow-up mortality between TAVR and sAVR groups. As Fig. 6 showing, though the total follow-up mortality was higher in TAVR (18.9%) than sAVR group (15%), pooled results showed no significant difference between TAVR and sAVR groups, with a pooled OR of 1.24 (95% CI 0.85–1.83; *P* = .27). In addition, subgroup analysis similarly indicated no significant difference in total follow-up mortality between TAVR and sAVR groups in CABG patients (OR 1.11; 95% CI 0.84–1.46). However, for patients previously received multiple cardiac surgeries, subgroup analysis indicated higher total follow-up mortality in TAVR than sAVR (OR 2.28; 95% CI 1.09–4.77) (Table 3).

**Figure 7:**
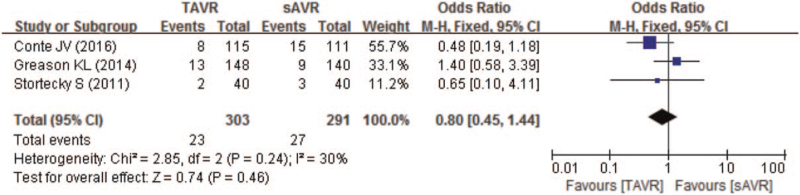
Forest plot of comparison between TAVR and sAVR regarding to cardiovascular mortality. sAVR = surgical aortic valve replacement, TAVR = transcatheter aortic valve replacement.

Pooled analysis showed no significant difference in CV mortality between TAVR and sAVR with a pooled OR of 0.80 (95% CI 0.45–1.44; *P* = .46) (Fig. 7).

### 3.4 Comparison between TAVR and sAVR regarding to clinical outcomes

We assessed the complications and other clinical outcomes between TAVR and sAVR for sever AS patients with PCS. Pooled results showed that, as a redo procedure, TAVR experienced higher pacemaker (PM) implantation than sAVR (OR 3.41; 95% CI 1.97–5.89; *P* < .0001). However, compared with sAVR, TAVR experienced shorter length of stay (MD –3.18 day; 95% CI –4.78 to –1.57) and procedural time (MD –172.01 min; 95% CI –251.15 to –92.88) respectively. TAVR also lead to much less bleeding than sAVR (OR 0.40; 95% CI 0.22–0.73; *P* = .003). No significant difference was found between TAVR and sAVR in stroke (OR 0.36; 95% CI 0.09–1.38) and acute renal failure (OR 0.80; 95% CI 0.30–2.16) (Table 4).

**Table 4:** The pooled results of comparison between TAVR and sAVR regarding to clinical outcomes.

		Pooled results			Heterogeneity		
Subgroups	No. of patients	MD/OR	95% CI	*P* value	*I* ^2^	*P*_h_ value	Analytical effect model
PM implantation	872	OR 3.41	1.97, 5.89	<.0001	21%	.28	Fixed effects model
Stroke	5579	OR 0.36	0.09, 1.38	.14	93%	<.00001	Random-effect model
LOS (day)	7704	MD -3.18	−4.78, –1.57	.0001	87%	<.00001	Random-effect model
ARF	8015	OR 0.80	0.30, 2.16	.66	94%	<.0001	Random-effect model
Procedural time (min)	834	MD -172.01	−251.15, –92.88	<.0001	99%	<.00001	Random-effect model
Bleeding	8115	OR 0.40	0.22, 0.73	.003	88%	<.00001	Random-effect model

ARF = acute renal failure, CI = confidence intervals, LOS = length of stay, MD = mean difference, OR = odds ratio, PM = pacemaker.

### 3.5 Publication bias

**Figure 8:**
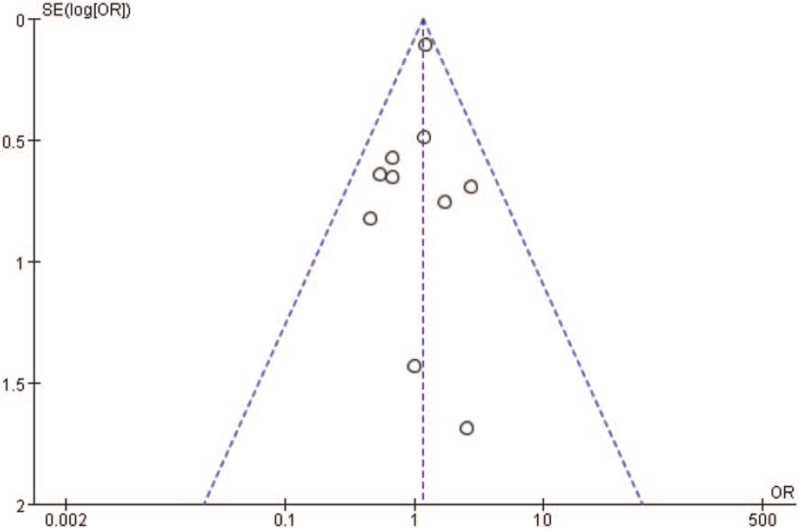
Funnel plot of comparison between TAVR and sAVR in 30-day / in-hospital all-cause mortality. sAVR = surgical aortic valve replacement, TAVR = transcatheter aortic valve replacement.

Begg funnel plot was generated to assess publication bias in the included studies. As shown in Fig. 8, the plots displayed no obvious asymmetry and showed no clear evidence of publication.

## 4 Discussion and conclusions

AS is one of the most common valvular problems associated with significant morbidity and mortality in the United States.^[31,32]^ The history of PCS was one of the high-risk factors for a redo procedure of cardiac surgery.^[33–35]^ This study aimed to evaluate the impact of PCS on clinical endpoints in patients undergoing TAVR versus sAVR as a redo procedure for severe AS.

Our pooled analysis of 8852 patients showed no difference in 30-day, 1-year, total follow-up all-cause mortality, and CV mortality between TAVR and sAVR for patients with a history of PCS. Regarding to baseline, compared with sAVR, TAVR group experienced a significantly higher STS score and older age patients. However, TAVR may lead to more PM implantation, but shorter length of stay and procedure time than sAVR. No difference in stroke and acute renal failure between the 2 groups was found. With consideration of the pooled results, we agree the advantage of TAVR as a redo procedure for patients with a history of PCS. Patients receiving TAVR experienced rapid recovery, shorter operation time, and less bleeding, without increasing short and long term mortality. Though our results indicated more PM implantation in TAVR groups, this result may be influenced by higher STS score and older age patients in TAVR groups, with may lead to any risk of bias to our results.

Previous study with 5 cohort studies (872 patients) explored this theme and found that patients with previous CABG who underwent TAVR had similar perioperative and long-term survival while experiencing more PM implantations and shorter hospital stay compared with those who had sAVR.^[36]^ However, the authors only included patients with previous CABG, and the number of studies was much too small, which may lead to any risk bias. We comprehensively explored influence of all PCS on AS patients receiving redo procedure. Our subgroup analysis indicated similar results for patients with previous CABG. However, our subgroup analysis found difference between TAVR and sAVR in subgroup of CABG, AVR, and mitral valve reconstruction. Reardon et al^[29]^ compared TAVR with SAVR outcomes in patients at intermediate operative risk with prior CABG surgery and came to a conclusion that both TAVR and sAVR were safe for intermediate-risk patients and the transcatheter approach facilitated faster improvement in quality of life and better exercise capacity at 1-year follow-up.

There existed several limitations in our work. First, due to lack of patient-level data, we could not perform additional subgroup analyses for other baseline characteristics. Though the baseline characteristics were comparable between TAVR and sAVR in included studies, studies have indicated that many population characteristics may influence the postoperative outcomes of patients. Second, there were noticeable variations among the studies with regard to the definition of surgical risk and outcomes, valve type, and delivery system. We failed to perform sub-group analysis to compare self-expanding and balloon expandable TAVR in previous cardiac surgery patients. As several studies were done with TAVR devices that are not contemporary, this review is limited to showing the effects of old TAVR devices. Winter et al^[37]^ conducted an overview on common complications related to the different TAVI devices and demonstrated a gradual improvement in peri-procedural mortality and complication with next generation devices as compared with first generation devices. Third, most of the studies included patients in the TAVR group who were deemed to be high risk with high Society of thoracic surgeons-predicted risk of mortality (STS-PROM) and European system for cardiac operative risk evaluation, putting them at higher risk for mortality and post-procedural complications. Fourth, limited to number of included studies, we failed to conducted subgroup analysis according to difference cardiac surgery, especially for CABG patients, such as on-pump or off-pump. Finally, we did not discuss the medical economics of the index procedures and health benefits measured as the number of added life-years or quality-adjusted life years. It was important to mention that the data regarding cost-effectiveness of TAVR (assessed by incremental cost-effective ratio for life-years or quality-adjusted life years) were more convincing for inoperable or high-risk candidates and predominantly favor affluent countries.

In conclusion, our analysis shows that TAVR as a redo procedure was equal to sAVR in mortality for severe AS patients with PCS, especially CABG. In addition, TAVR as a redo procedure may be favorable in reducing the incidence of bleeding, length of stay, and procedural time. However, TAVR may increase the incidence of PM implantation. Thus, we agree the advantage of TAVR as a redo procedure for patients with a history of PCS. However, future propensity score matched (PSM) studies should be conducted to compare the advantage of TAVR to sAVR avoiding many risk factors of baseline of patients.

## Author contributions

The authors on this paper all participated in study design. All authors read, critiqued, and approved the manuscript revisions as well as the final version of the manuscript. Also, all authors participated in a session to discuss the results and consider strategies for analysis and interpretation of the data before the final data analysis was performed and the manuscript written. All authors have the appropriate permissions and rights to the reported data.

**Conceptualization:** Jige Dong.

**Data curation:** Jige Dong.

**Methodology:** Guobin Wang.

**Software:** Guobin Wang.

**Writing – original draft:** Guobin Wang, Zhaojie Zhang.

**Writing – review & editing:** Guobin Wang, Zhaojie Zhang.
